# Evaluating Bempedoic Acid for Non-alcoholic Fatty Liver Disease: A Review of Preclinical and Clinical Research

**DOI:** 10.7759/cureus.67151

**Published:** 2024-08-18

**Authors:** Mohamed Anasdeen S, Shahinaz S, Vijayakumar TM

**Affiliations:** 1 Department of Pharmacy Practice, College of Pharmacy, SRM Institute of Science and Technology, Chennai, IND

**Keywords:** non-alcoholic steatohepatitis (nash), non-alcoholic fatty liver disease, low-density lipoprotein (ldl) cholesterol, metabolic disorder, bempedoic acid

## Abstract

Non-alcoholic fatty liver disease (NAFLD) is the most prevalent liver disorder globally, characterized by fat accumulation in liver cells, which can progress to inflammation, fibrosis, and cirrhosis. The disease predominantly affects individuals with obesity and high body mass index (BMI). It is a globally prevalent condition, with variations in incidence across different regions. The pathophysiology of NAFLD involves insulin resistance, metabolic disturbances, and genetic and gut microbial factors. Current treatments primarily focus on lifestyle modifications and a limited range of pharmacological options. Bempedoic acid (BA), a novel cholesterol-lowering agent, targets adenosine triphosphate (ATP)-citrate lyase to reduce low-density lipoprotein (LDL) cholesterol and has shown potential in managing NAFLD by decreasing liver fat accumulation and improving lipid profiles. BA's unique mechanism offers a promising add-on therapy, particularly for the patient’s intolerant to statins. Despite its potential, comprehensive clinical and preclinical studies are needed to further elucidate its efficacy and safety compared to other NAFLD treatments. Future research should focus on comparing BA with existing and emerging therapies to optimize its role in NAFLD management and enhance patient outcomes.

## Introduction and background

Non-alcoholic fatty liver disease (NAFLD) is the most common liver disease around the world. It is a chronic condition where fat accumulates in liver cells, leading to inflammation and pain with few or no symptoms in the early stages. As the disease advances, it can cause increased pain in the upper abdomen and may develop into non-alcoholic steatohepatitis (NASH), which can result in fibrosis and cirrhosis. Individuals who are overweight or with a body mass index (BMI) greater than 33 kg/m² are more likely to develop NAFLD according to the World Health Organization. The epidemiology of liver cirrhosis has been thoroughly examined in several developed nations across Europe and America. In many developing countries, the lack of sufficient data on the disease has led to less attention being paid to its mortality rate. The global health community is increasingly recognizing the need to manage risk factors for liver cirrhosis, particularly alcohol use and chronic infections with hepatitis B and C viruses. In its early stages, NAFLD exhibits no noticeable symptoms. As the illness worsens, though, people may feel fatigued, irritable, or have pain in their upper right abdomen. They can also develop an enlarged liver. Unexpected weight loss may also be seen by certain patients. In severe cases, NAFLD can lead to NASH, which can further lead to cirrhosis and decompensation. Early detection and management of NAFLD are crucial to prevent severe liver damage, cirrhosis, and liver dysfunction. NAFLD has a wide range of complex causes. The fundamental cause is insulin resistance, which is the body's cells' poor response to insulin, resulting in the accumulation of fat in the liver. This is often related to lifestyle factors; obesity, particularly excess weight around the abdomen, and diseases such as type 2 diabetes and high cholesterol are major contributors. Consuming excess sugar, processed carbohydrates, and unhealthy fats can cause fatty liver. Recent studies state that gut bacteria have a slight impact on fat accumulating in the liver [1]. Bempedoic acid (BA) is a cholesterol-lowering drug that blocks an enzyme called adenosine triphosphate (ATP) - citrate lyase - which helps produce cholesterol. Through this process, BA lowers cholesterol levels. Some studies show that BA can help prevent NAFLD from progressing to NASH [2].

## Review

Pathophysiology

**Figure 1:**
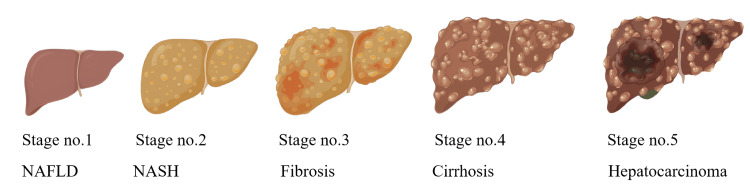
Progression of the degrading liver. This image was inspired by the article by Bessone et al. (2019) and was recreated by the author [3].

The primary cause of NAFLD is overnutrition, which results in the accumulation of fats in adipose and non-adipose tissues. Mostly due to insulin resistance, which results in uncontrolled lipolysis, which significantly reduces the liver's metabolic capacity. This imbalance results in metabolism leading to harmful lipids causing inflammation, lipid accumulation, and even cell death. Macrophages play a significant role in liver fibrosis, which results in chronic inflammation in liver tissues. The exact mechanism behind NAFLD is still unknown but the most predominant factors influencing NAFLD are metabolic, genetic, and gut microbial conditions. Genetic factors alone contribute up to 30-70% in disease risk based on the heritable component. Alteration in gut microbes can have a significant effect on NAFLD because of their vital role in lipopolysaccharides, and bile acid production which may contribute to inflammation in the liver and disease progression. Inflammatory responses, oxidative stress, and genetic predisposition are among the key factors contributing to the development of NAFLD. Additionally, metabolic disturbances, such as insulin resistance and dyslipidemia, along with lifestyle factors such as poor diet and sedentary behavior, further exacerbate the condition. Figure 1 represents the progression of the degrading liver due to NAFLD [3].

Current treatment landscape

Non-pharmacological Management

Lifestyle changes, including adjustments to diet, exercise, and physical activity, are fundamental in managing NASH. The main objectives of these approaches are to control metabolic conditions and maintain a healthy body weight through methods, such as calorie restriction, time-restricted eating, and regular physical exercise.

Pharmacological Management

Pharmacological treatment is generally considered to address the underlying causes and to improve the over conditions. In patients with NAFLD, insulin sensitivity is the main and most common reason for the accumulation of fats in the liver. Many studies have been conducted and proven by Ratziu et al. [4] and Aithal et al. [5] that enhancing insulin sensitivity through administering drugs in the classes of thiazolidinediones improves insulin sensitivity in patients with NASH. A study conducted by Wang et al. [6] on vitamin E supplements proved that it had significant improvement in liver enzyme levels and reduced fibrosis in the long term. Yoon et al. [7] conducted a study on a combination of ursodeoxycholic acid and vitamin E in patients with NAFLD, and they recorded a significant improvement in liver recovery [7]. Research was conducted by Younossi et al. [8] on the use of obeticholic acid to treat NASH, and the findings indicate a noteworthy improvement in the patient's fibrosis and recovery [8]. A new investigation drug called tropifexor agonist of non-bile acid farnesoid X receptor inhibits bile acid synthesis and increases excretion, which ultimately results in less bile accumulation in the liver; hence, this drug is in its phase 2 trials and is expected to be used for the treatment of NASH [9]. Another promising drug treatment for NAFLD is peroxisome proliferator-activated receptors (PPAR) agonists, which are elafibranor and saroglitazar. Elafibranor showed a 19% NASH resolution without worsening fibrosis compared to 12% for placebo in a phase 2b trial [10]. As of the phase 3 results reported, elafibranor clearly has advantages in lowering the risk of NASH progressing to fibrosis or cirrhosis, but it has recorded many side effects in the trials, including abdominal pain, diarrhea, nausea, and vomiting [10]. Gawrieh et al. [11] conducted a 16-week trial with 106 NAFLD/NASH patients. Saroglitazar significantly reduced alanine transaminase (ALT) levels compared to a 3.4% increase with placebo. The 4 mg dose also improved liver fat content, adiponectin, insulin resistance, and triglycerides. Saroglitazar was well-tolerated, with a mean weight gain of 1.5 kg compared to 0.3 kg with placebo [11]. An oral THR-β agonist known as resmetirom received accelerated approval in March 2024 for the treatment of noncirrhotic NASH with moderate to advanced fibrosis in the United States. The drug is currently under review for regulatory approval in the European Union [12]. These are the most common and novel pharmacological treatments, which are in clinical practice, and few of them in clinical trials for the treatment of NAFLD and NASH.

Difference between BA and other drugs used in NAFLD

BA is a new medication primarily known for its lipid-lowering effects, particularly in reducing low-density lipoprotein (LDL) cholesterol levels, but it shows some promise in the management of NAFLD. The key mechanism of BA is inhibiting ATP-citrate lyase, which helps reduce hepatic fat accumulation and improve lipid profile. This has an indirect effect on patients with NAFLD. The mechanism of BA is different from other drugs commonly used for NAFLD. In contrast with pioglitazone, thiazolidinediones improves the sensitivity of insulin, which results in a direct effect on liver fat reduction. It is often limited by its side effects, including weight gain [4,5]. Obeticholic acid, a farnesoid X receptor agonist, largely targets bile acid metabolism, which has been shown to reduce inflammation and fibrosis in NASH patients rather than focusing on lipid metabolism [8]. Another drug liraglutide, a glucagon-like peptide 1 (GLP-1) receptor agonist primarily used for diabetes, helps improve insulin sensitivity by promoting weight loss and shows some promise in treating NAFLD. BA can be used as an adjuvant therapy along with other drugs for the effective treatment of NAFLD [13].

Clinical efficacy and effectiveness

The clinical efficacy of BA for the treatment of NAFLD is still being explored. Here is a summary of the current understanding.

Lipid-Lowering Effect

BA primarily acts as a lipid-lowering agent by inhibiting ATP citrate lyase enzymes, leading to reduced LDL levels and cholesterol levels. This can indirectly have a superior effect on patients with NAFLD and NASH, especially with synchronic dyslipidemia. As the lipid profile improves, fat accumulation and overall metabolic syndrome reverses in the early stages [2].

Indirect Liver Benefits

BA reduces the fat content in the liver, by lowering cholesterol and LDL levels, which is an important aspect of NAFLD management. A few preclinical and clinical studies are listed below, which will provide a comprehensive outline of the efficacy of BA in the management of NAFLD.

Complementary Effectiveness

BA is commonly used in combination therapy with other lipid-lowering agents, including statins and non-statins lipid-lowering drugs. It is predominately used in patients with statin intolerance. It is more effective as an adjuvant therapy rather than monotherapy.

Safety Profile

It has a well-tolerated effect with a good safety profile. As an added advantage, it is a hydrophilic drug; hence, it has very little to zero effect on the liver, making it very compatible with treating patients with chronic conditions such as NAFLD and dyslipidemia [3].

Preclinical studies

The efficacy of BA in preclinical studies for NAFLD is still in the early stages. Animal models have shown promising effects on reducing liver fat and improving lipid metabolism. These studies indicate that BA decreases de novo lipogenesis and lowers hepatic fat accumulation in animal models. Table 1 provides an overview of BA's effectiveness in animal models.

**Table 1:** List of pre-clinical studies on the effectiveness of BA.

S.no	Author	Animal	Diet	Dose per day	Days	Observation	Ref
1.	Desjardins et al.	C57BL/6J mice	High-fat high-fructose diet	10 mg/kg of BA and 70 µg/kg of liraglutide	112	The combination reduced up to 44% of fibrosis compared to liraglutide and a significant reduction in triglycerides and cholesterol	[13]
2.	Samsoondar et al.	Mice	High fat-containing diet	3 mg/kg 10 mg/kg 30 mg/kg	84	10& 30 mg/kg batch has significantly less cholesterol & triglycerides compared to the control group and liver enzymes	[14]
3.	Burke et al.	Yucatan miniature pigs	High-fat high-calorie diet	120 mg/day 240 mg/day	160	It has a significant decrease in plasma lipid concentration up to 60% without altering the liver enzymes	[15]
4.	Pinkosky et al.	Mice	High-fat high-calorie diet	30 mg/kg	84	It showed a remarkable difference in the lipid levels with no alteration in liver enzymes	[16]

Clinical studies

NAFLD is a widespread global health issue, and recent research highlights the potential of BA in addressing this condition. The data presented in Table 2 demonstrate that BA, whether used alone or in combination with other agents, has achieved remarkable results in the management of dyslipidemia. This evidence suggests that BA is an excellent option for patients with NAFLD who are intolerant to statins, offering a viable alternative with relatively low toxicity. Its efficacy in improving lipid profiles while maintaining a favorable safety profile underscores its promising role in the comprehensive treatment of dyslipidemia and NAFLD.

**Table 2:** List of clinical studies on BA with placebo and other drugs.

S.no	Author	Groups + dose in mg								Total population	Ratio	Weeks	Observation	Ref
1.	Ballantyne et al.	Bempedoic acid (180)				placebo				269	2:1	12	Significant decrease in LDL-C, apolipoprotein B, and C-reactive protein levels	[17]
2.	Ballantyne et al.	BA+EZE (180+10)			EZE (10)	BA (180)	PO			301	2:2:2:1	12	BA+EZE produced a significantly greater effect which then followed BA alone reduced LDL-C levels.	[18]
3.	Thompson et al.	BA (120)	BA (180)		BA+EZ (120+10)	BA+EZ (180+10)	EZ (10)		PO	322	4:4:4:4:1:1	12	48% reduction in LDL levels by BA+EZ (180mg) shows the significance of BA.	[19]
4.	Gutierrez et al.	BA (80)			PO	BA (120)	PO			60	1:1:1:1	4	BA (120mg) reduced 43% LDL-C & 38% in BA compared with placebo	[20]
5.	Shapiro et al.	BA in nonmetabolic disease		PO in non-metabolic disease		BA in metabolic disease		PO in metabolic disease		2509	6:4 & 7:3	52	BA in non-metabolic disease reduced LDL-C even in metabolic disease it produced safe results without elevation of liver enzymes	[21]
		1037		536		648		288						

Future direction and research

Although existing research provides valuable insights, the current body of evidence available online is limited and does not encompass all aspects of BA's efficacy and safety. To gain a more comprehensive understanding of its potential, further research is essential. Conducting additional clinical studies and comparing BA with other dyslipidemic and NAFLD treatments will be crucial in elucidating its benefits and limitations. Such comparative analyses will help establish a clearer picture of BA's role in managing these conditions and guide its future applications in clinical practice.

## Conclusions

In summary, NAFLD represents a significant global health challenge with complex etiologies and varying regional prevalence. While lifestyle modifications and existing pharmacological treatments offer some relief, the management of NAFLD and its progression to more severe conditions such as NASH remains a critical area of focus. BA, a novel lipid-lowering agent, has shown promising results in improving lipid profiles and potentially mitigating liver fat accumulation, presenting a viable option, especially for patients intolerant to statins. Despite the encouraging data, the full potential of BA in NAFLD management is still under investigation. There is a pressing need for further clinical research to better understand its efficacy and safety, particularly in comparison to other NAFLD and dyslipidemia treatments. Continued exploration and well-designed studies will be crucial in determining the optimal role of BA in the therapeutic landscape and ensuring it contributes effectively to the comprehensive management of NAFLD.
